# Mandibular reconstruction using single piece zygomatic implant in conjunction with a reinforcing Fibular Graft Union: A case report

**DOI:** 10.1016/j.ijscr.2020.07.047

**Published:** 2020-07-23

**Authors:** Vivek Gaur, Anita Gala Doshi, Lukasz R. Palka

**Affiliations:** a Jaipur Dental College, Maharaj Vinayak Global University, Jaipur, India; b Dentahealth Clinic, Mumbai, India; c Reg Med Clinic, Zary, Poland

**Keywords:** Zygomatic implant, Single piece bicortical implant, Immediate Loading, *Mucosalization*

## Abstract

Presenting to our knowledge, for the first time in literature, a case report on the long term follow-up of an implant retained fixed prosthesis on free fibular reconstruction with a single piece zygomatic implant and single piece bicortical implant via a flapless approach and immediate functional rehabilitation. The technique involved the concept of “Remote Bone Anchorage” in conjunction with the union of grafted free fibula flap with the native mandible. It was facilitated with a single piece zygomatic implant engaged in the mandible and splinted with single piece implants on the grafted fibula as needed for prosthetic functional reconstruction. The author has reported a unique concept of immediate functional rehabilitation in the fibular graft therefore providing additional splinting of the vascularized free fibula to the mandible by splinting them with single piece zygomatic cortical implant.

## Highlights

• This study reports about the success in regard to long term follow-up of an implant retained fixed prosthesis on free fibular reconstruction with a single piece zygomatic implant.
• It also reports the superiority of using a single piece bicortical implant via a flapless approach and immediate functional rehabilitation.
• Remote Bone Anchorage in conjunction with the union of grafted free fibula flap with the native mandible.
• Moreover, it demonstrates the long term success of the technique of immediate functional rehabilitation in the fibular graft.

## 1 Introduction

Mandibular ablative surgeries consist of enucleation, marginal resection, segmental resection or en-bloc excision of the complete jaw. Often, extirpation of a pathologic tissue that may be benign or malignant is performed by ablative surgery. This may result in defects such as improper articulation, facial asymmetry/deformities, difficulty in deglutition, speech and breathing difficulties, impaired sensory and motor control of tongue, drooling of saliva from the corner of mouth and mastication that have a functional, aesthetic and psychological impact on the patient, thus creating a need for it to be reconstructed surgically as a treatment of choice as well as a treatment option for post traumatic cases. The concept of surgical reconstruction aims to close the defect that results from the ablative procedure and the restoration of lost function. If post resection radiotherapy is advised, free vascularized osseous or osteocutaneous grafts are preferred [1,2].

Successful jaw rehabilitation requires the restoration of bone continuity, height and bulk, dental arch form so that a fixed or removable implant borne prosthesis can be delivered [3]. The reconstruction of lower lip sensation in mandibular reconstruction is now a standard procedure by microneural anastomosis. Before the advent of microvascular free flaps, such patients with post ablative surgeries used to be referred to as “forgotten patients” [4]. The fibular free flap and Iliac Crest free flap with internal oblique are the two most common vascularized myocutaneous-osseous graft preferred for the mandibular reconstruction. The fibular flap has an available dipole/bicortical bone upto 25 cm–27 cm length with a pedicle based on peroneal artery and vein, which is composed of 2–3 mm diameter and around 15 cm length allowing the anastomosis with branches of the external carotid artery. In 1989, Hidalgo became the first person to transfer fibular bone to reconstruct a segmental defect of the mandible [5], but Taylor et al. [6] used this method to reconstruct the tibia in 1975. The fibular bone that contains endosteal and periosteal blood supply allows the osteotomization in multiple sections to get adapted for the desired shape as well as allows it to resist atrophy better than the native jaw bone as the native mandible is intra-membranous in origin compared to fibular bone which is endochondral in origin [7].

Implants may be placed at the time of ablative and reconstructive surgery [primary implants] or at a later date [secondary implants] [8]. Immediate functional loading of the prosthesis on the fibular construction is rare and has been published only once [9]. Studies have reported of the success of rough surface two stage conventional implants in the fibular flap reconstruction. The smooth surface single piece zygomatic cortical implant are the tool of choice for the rehabilitation of the reconstruction jaws, with or without vascularized flaps [10]. They are in indeed the **Oncology Implants**. For the two stage implants the limitation of fibula diaphysis is corrected by techniques like double barrel fibula reconstruction or the distraction osteogenesis following Illizarov principle [11] to overcome unfavorable crown root ratio. But the technique itself have their own limitations. It’s evident that the marginal bone loss is higher around moderately rough surface implants when compared to turned/smooth surface implants [[12], [13], [14]].

Hence, this case reports the rehabilitation by implant retained fixed prosthesis in free fibular reconstruction with single piece zygomatic implant by flapless approach and immediate functional rehabilitation. The aim was to provide rehabilitation with implant borne prosthesis using a fixed prosthesis along with a Flapless single piece zygomatic implant placement thus engaging the other cortical of fibula graft for high insertion torque and thereby allowing the application of zygomatic single piece implant to engage into the left symphysis region of the native mandible as remote implant anchorage [14] and splinting with other implants and creating an additional splinting of the fibular graft to native mandible (a unique and first of its kind concept/approach).

## 2 Case report

A 45 years old male patient presented with a history of resection surgery of the right side of the body of the mandible for the extirpation of lower buccal right side squamous cell carcinoma lesion along with functional neck dissection two years back. The defect was reconstructed by a vascularized free fibula graft with the skin island covering the intra oral defect successfully along with tumor ablation surgery done simultaneously. The patient was administered therapeutic radiotherapy post ablative surgery. Maxillary complete dentition was present and in the lower left jaw, canine until the left second molar was present. Right side free fibula graft was present stabilized by osteosynthesis plates. There was excess of skin flap over the fibula graft restricting the inter-arch space with opposite dentition and was difficult to occlusally rehabilitate with prosthesis. There was no vestibular sulcus and mucosa with restricted tongue movement because of lingual space occupied by skin flap. In the radiograph and CT scan, it was evident of the space present between the fibula and the symphysis indicating of delayed union/non-union.

1. Pre operative panorama

**Figure:**
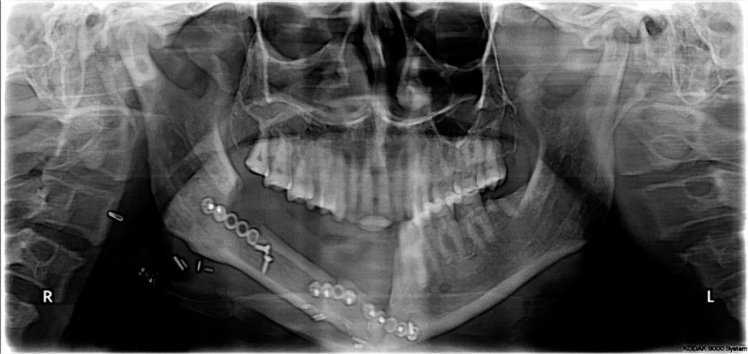

2. Pre operative computerized scans

**Figure:**
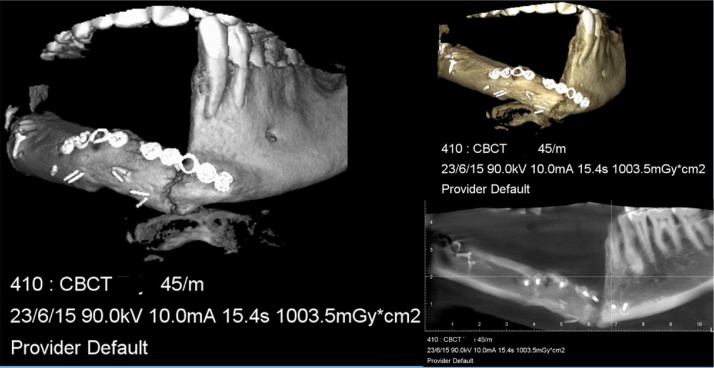

3. Intra oral picture

**Figure:**
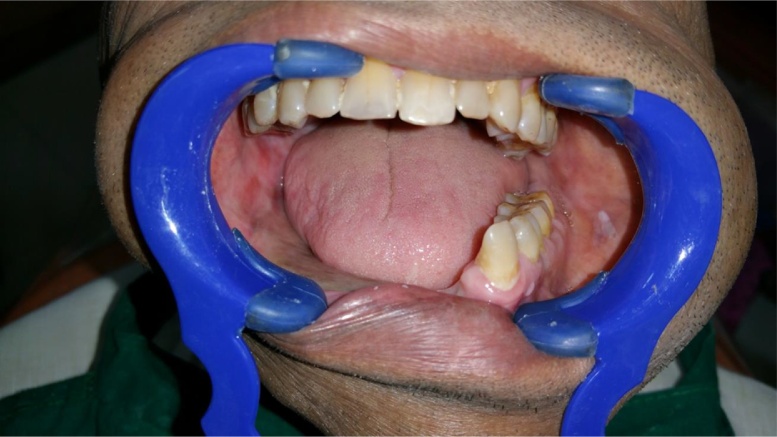

For successful prosthetic rehabilitation, there is a need to understand and evaluate the extent of the defect and its limitations. Various classifications for evaluation of mandibular defects have been described such as Jewer’s classification of Mandibular defects [15] Boyd classification of mandibular defects [16], Urken et al. [17] Classification of mandibular defects [18], Cantor and Curtis classification of mandibular defects [[19], [20], [21]], but the most latest and simplified one reported is by James S Brown et al. The limitation of James S Brown et al. [22] classification is that soft tissue and type of dentate status has not been described. Our paper presented a class 2 defect of James S Brown classification.

### 2.1 Surgical procedure

After the routine blood examination and written consent was obtained from the patient, the patient was operated in a routine dental operatory under local anesthesia. Lignox® 2% A [lignocaine with adrenaline 1:80000] was infiltrated around the fibula. Following the manufacture instructions, five BECES® implants [Manufacturer: Simpladent GmbH, Switzerland] and one ZDI® implant [Manufacturer: Simpladent GmbH, Switzerland] were placed via a flapless procedure. From distal to mesial, the dimension of BECES® implants engaging the lower cortex of the fibula were of 3.6 mm diameter but length of 29 mm, 26 mm, 29 mm, 26 mm and 23 mm. The ZDI® implant of 4.6 mm diameter and length 35 mm was angulated towards the left symphysis from the mesial aspect of fibula flapless and bended by AHB® adapter for the restorative acceptable position. The placement of implants were itself a challenging task as the implants were long and limited inter arch clearance resulted in difficulty of the implant insertion. There were more than 10 mm of the dead space because of mobile skin island over the fibula.

### 2.2 Prosthetic procedure

Impression was made on pickup impression caps supplied along with implants by polyvinyl additional silicon putty impression material (Aquasil® - dentsply), the same day after the implant placement. Next day metal framework fit was checked over the implant abutment intraorally and in the evening metal to acrylic semi-permanent hybrid prosthesis was delivered keeping the occlusion lingually inclined. The surgeon skillfully placed all implants free hand avoiding the osteosynthesis screws present for the fibula fixation.

### 2.3 Follow up

After a year, the prosthesis was removed by cutting the metal framework carefully by carbide burs and all implants were checked for the stability. One loose implant was found to be in contact with the osteosysnthesis screws and thus failed to osseointegrate and was hence, removed. The prosthesis was refabricated keeping sanitary intaglio profile. Tissue around the implants were asymptomatic with no complaints from the patient. The surgeon was successful in achieving the union of the fibula to native mandible and was evident radiographically. On three years follow up, the patient complained of some growth without pain around the neck of the implants on the transplanted fibula. A biopsy was then performed that reported negative. On examination, it was observed that complete *mucosalization* of the skin graft had occurred with no observable distinction from the mandibular mucosa of the patient. It was concluded that the growth was the granulation tissue around the implants which was then excised by soft tissue 810 nm laser with 400 μm fibre. Presently, the patient is completely satisfied and doesn’t have any complaint up till now and there is a healthy mucosa all around the intaglio surface of the functional prosthesis.

4. BECES® and ZDI® implants placed flapless

**Figure:**
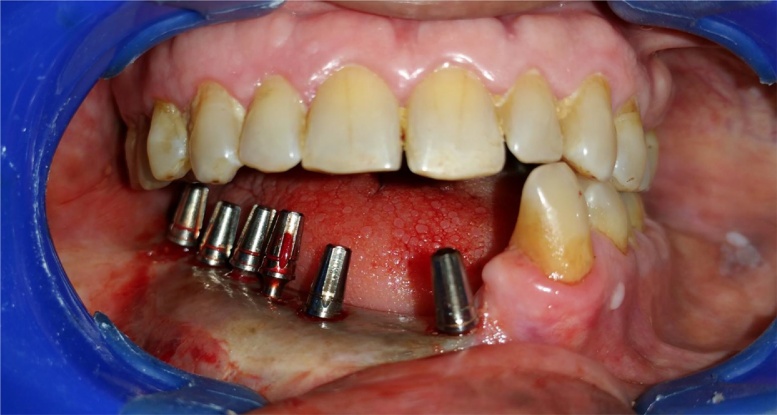

5. Post operative panaorama

**Figure:**
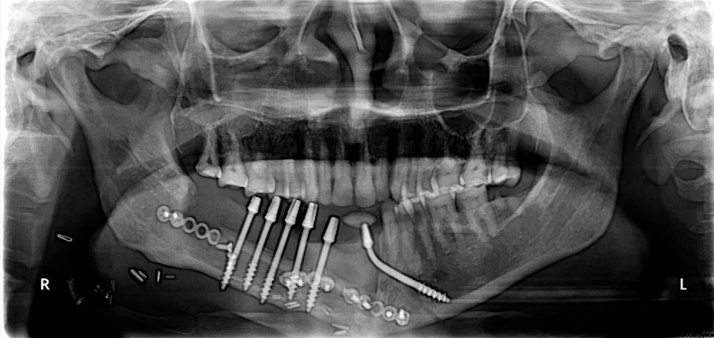

6. One year follow-up

**Figure:**
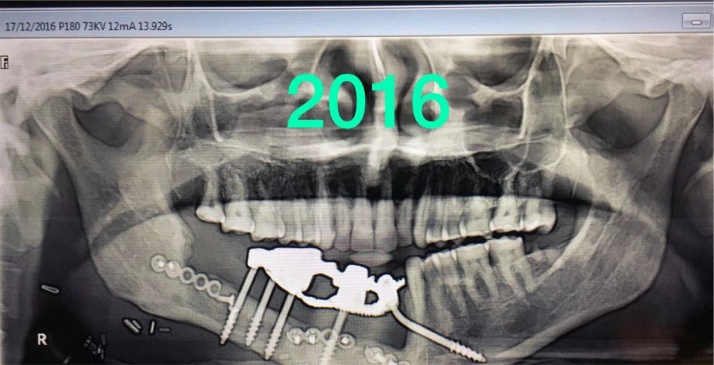

7. Two year follow-up

**Figure:**
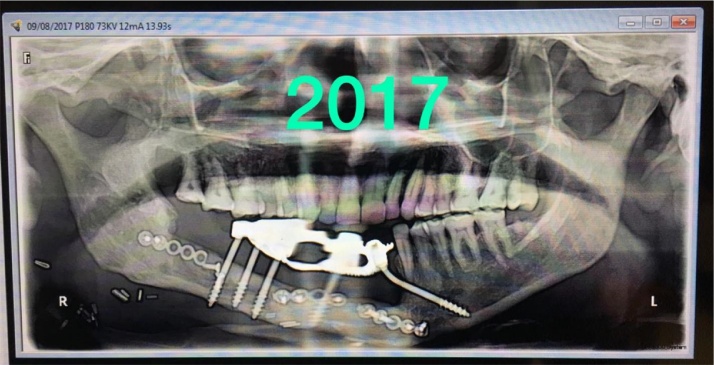

8. Three year follow-up up to 04/2108

**Figure:**
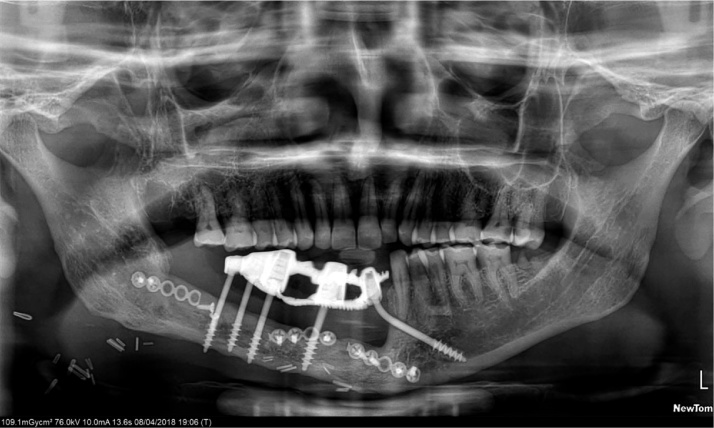

9. Growth around the implant neck

**Figure:**
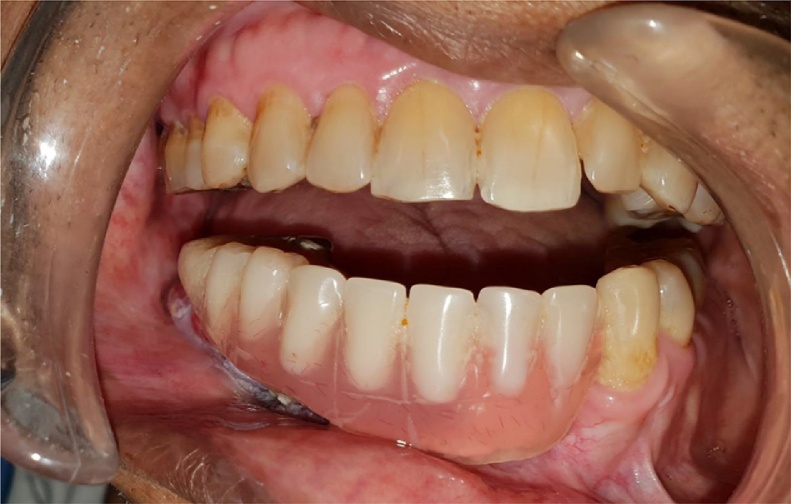

10. Granulation tissue excised by soft tissue 810 nm laser

**Figure:**
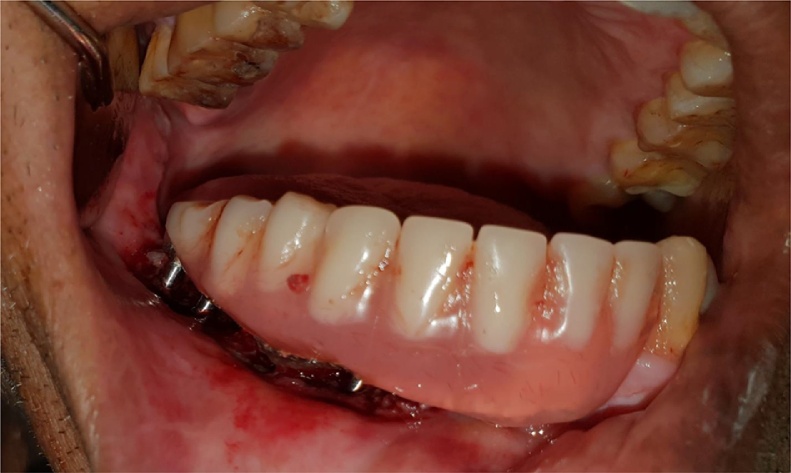

11. Healthy mucosalized tissue appreciated with sanitary prosthesis

**Figure:**
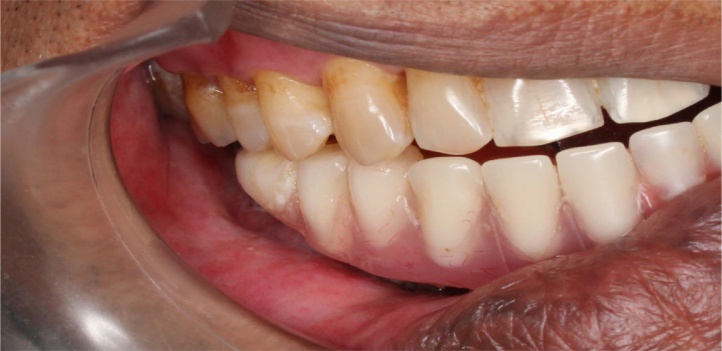

## 3 Discussion

In our study, we report for the first time, the mandibular reconstruction by an implant retained fixed prosthesis on free fibular reconstruction with a single piece zygomatic cortical implant. The fibular graft is called the workhouse of mandibular reconstruction. It’s touted as the most donatable bone in the body [23]. Among all the free flaps, implants on the fibula have the highest success as being most osteogenic. The fibula is resistant to crestal bone resorption since its cortical bone has high content of osseous morphogenic proteins, which act osteoinductively, promoting the bone healing process [[24], [25], [26]]. Fibular flap shows a long term ability to maintain mass over time, with the rate of atrophy significantly lower then the native bone, mandible [27,28]. The sural nerve with fibular flap is harvested and grafted into the defect by microneural anastomosis to the proximal and distal stump of the inferior alveolar nerve. But it should loop around the lower border to facilitate dental implant placement. Skin island/paddle is vascularized by septocutaneous perforators from the peroneal artery, can be used both for intraoral and extraoral closure. The graft can be harvested intraoperatively at the same time of resection surgery. The limitation of the free fibula diaphysis is of 14 mm, the skin graft is coarse, thick, and sinking to about 1 cm under compression [29] over the fibular bone and the post-operative morbidity of the donar site resulting in insufficient gait restoration and difficulty in walking during complex task and at high velocity. The insertion of multiple screws and plates to ensure fixation did not appear to affect bone volume. Care is to be taken not to cut the segments smaller then 3 cm as advised by Schrag et al. [30].

We used smooth surface single piece implants (Strategic Implant®) and although rough surface implants have reported better success over the smooth surface counterparts in fibular grafts, the studies mentioned do not report follow-ups. And often, implant success is confused over implant survival rate [31]. There is continuous bone resorption and increase in pocket depth. When exposed to the oral environment, implants with rough surface may facilitates the accumulation of plaque affecting the equilibrium with the host [32,33]. Consequently, implants with rough surface loose more bone when compared to implants with turned surfaces [34]. Patient acceptability and comfort is highest with smooth surface basal implants [35,36]. Betz et al. reported a mean probing depth of 5.1 mm in tumor patients, as against 3.4 mm in non-tumor patients. Investigations state that the incidence of peri-implant soft tissue inflammation and pocket depths increase over time. However there is an *adaptive rebuilding* phenomenon proposed by Kovacs [35], takes place with transplanted soft tissue despite of the plaque accumulation. This rebuilding leads to leads to a decrease of peri-implant inflammation [37] over time despite of implants surface surrounded by movable [non passive] soft tissue. In the cases of completely implant-supported prosthesis there is possibility of elastic adaptation [38]. The increased numbers of micro-organism colonies around the implants probably associated with trigger factors like loose abutment or micro gap within a fixed restoration which in cases are common with rough surface two stage conventional implants. Exposure to oral contaminants is more common at percutaneous sites because of this non existing barrier between the skin and the implant [38,39].

The characteristic success of smooth surface single piece Strategic Implant® in the case presented was the *mucosalization* achieved with the functional stability of the prosthesis which was immediate functional loaded in vascularized free fibula flap. *MUCOSALIZATION* is change from skin to mucosa observed in skin flaps [40] applied in oral cavity with respect to color, desquamation and mucosa like shape. The epithelium show parakeratosis with no melanin pigmentation or epithelial pegs. In the subepithelial connective tissue, few capillaries, fibroblasts and collagen fibers remain. The hair roots, hair follicles and sebaceous glands all disappear [39]. The change in environment which is moisture laden with saliva intra-orally and change in bacterial flora, mechanical and chemical stimuli associated with food ingestion and candida infection leads to successful *mucosalization*. There is secondary intention healing beneath the *mucosalized* skin graft results in granulation tissue formation which was evident in the current case report [41].

## 4 Conclusion

The case presented above, has produced results with high success for immediate functional prosthetic restoration of vascularized fibula flap based reconstruction case by single piece zygomatic cortical implant with very high acceptation from the patient as the results were achieved with a flapless protocol in minimal time and fewer appointments in the most economical way with a very desired predictable result. The union of fibula and the mandible was achieved with success following *Remote Implant Anchorage* concept with single piece zygomatic implant. The author proposes to place Single piece smooth surface bicortical implant in a new category of *Oncology Implant*.

## Conflict of interest

No conflicts of interest.

## Sources of funding

No funding.

## Ethical approval

N/A.

## Consent

Written informed consent was obtained from the patient for publication of this case report and accompanying images. A copy of the written consent is available for review on request.

## Author contribution

Dr. Vivek Gaur contributed to the conceptualization, validation, managing the patient, writing the manuscript.

Dr.Anita Gala Doshi contributed to the conceptualization, validation, editing and finalization of manuscript.

Dr. Lukasz Palka contributed to the conceptualization, validation, editing and finalization of manuscript.

## Registration of research studies

N/A.

## Guarantor

Dr. Vivek Gaur.

## Provenance and peer review

Not commissioned, externally peer-reviewed.
